# Supplementary material for: Are carbon emissions trading and green financial instruments synergistic? -Comprehensive quantitative research based on content analysis
Source: PLoS One. 2024 Mar 7;19(3):e0298601. doi: 10.1371/journal.pone.0298601 (PMC10919601; doi:10.1371/journal.pone.0298601)
Supplement: S1 Data — (ZIP) [file pone.0298601.s001.zip › Data/地级市Z_F工作报告.zip!/╣π╢1⁄2/╤⌠╜¡╩╨/└┤╫╘╣1⁄2╓┌║┼í╛┬φ┐╦╩2╛▌═°í┐_2013.docx]

各位代表：

　　我代表市人民政府向大会作工作报告，请予审议，并请市政协委员和列席人员提出意见。

　　2012年工作回顾

　　2012年是本届市政府施政的第一年。一年来，在省委、省政府和市委的正确领导下，在市人大及其常委会和市政协的监督支持下，市政府团结带领全市人民，紧紧围绕“在加快发展中转变方式、实现幸福追赶”的核心任务，全力稳增长、调结构、惠民生、抓改革、促和谐，全市经济社会保持良好的发展态势，主要经济指标增幅居全省前列，较好地完成了市六届人大一次会议确定的目标任务。

　　——经济实力稳步提升。初步核算，全市地区生产总值实现880亿元，增长13 %；人均生产总值35800元，增长12 %；规模以上工业总产值突破1000亿元，达1181亿元，增长23.3%；地方公共财政预算收入43.1亿元，增长23.4%；外贸进出口总额22.8亿美元，增长6%；城镇居民人均可支配收入19072元，增长13%；农村居民人均纯收入9415元，增长19%；单位生产总值能耗控制在省下达指标范围内。工业加快发展。全市规模以上工业增加值 282亿元，增长23.9%；最高供电负荷突破100万千瓦，达108.5万千瓦，增长25.4 %。重工业总产值增长34.4%，增速比轻工业高20.8个百分点。传统产业转型升级步伐加快，自主创新能力稳步提升，市本级科技三项经费增长22%，新增省级工程技术研究开发中心1家，阳江餐厨用品基地被认定为国家级外贸转型升级专业型示范基地，“中国陶瓷刀中心”和“中国打火石中心”先后落户阳江，五金刀剪产业总产值316亿元，增长13%。民营经济蓬勃发展，规模以上民营工业总产值682亿元，增长20.4%，在年度全省民营经济考核中名列第二。高效农业持续发展。农业总产值实现285亿元，增长4.9%。创办8个国家级万亩水稻高产示范片，粮食总产量73.8万吨，实现了“四连增”。建成现代农业园区40个、优质水果标准化生产示范基地3.2万亩，南药、花卉等特色经济作物种植295.3万亩。远洋渔业生产取得突破，首个深水网箱产业园投苗生产；引进了南美白对虾亲本核心技术；东平国家级中心渔港建设加快，沙扒国家一级渔港一期工程顺利竣工，渔业总产值115.4亿元，增长6.7%，水产品出口货值5.8亿美元，在全省保持领先。第三产业提速发展。完善了海陵旅游发展总体规划，举办了以“碧海蓝天·船说阳江”为主题的旅游文化节和第十届南海（阳江）开渔节，评选了“2012年阳江十大最美乡村”，推进了十里银滩综合开发等旅游重点项目建设，全市新增4A和3A旅游景区各1个，接待游客总人数1085.5万人次、旅游总收入86.4亿元，分别增长20.7%和30.2%。汇丰银行入驻我市，填补了阳江外资银行的空白；全市金融机构年末存贷款余额分别比年初增长13.6%和20.4%。商贸物流等现代服务业稳步发展，社会消费品零售总额470亿元，增长15%。

　　——重点项目建设成效明显。固定资产投资完成473亿元，增长18%。年产10万吨纯镍合金等5个重点项目建成投产，阳江核电站等27个续建重点项目加快推进，阳阳高速至S277连接线工程等12个重点项目开工建设，阳江抽水蓄能电站等4个重点项目获国家批准开展前期工作。跑资金争项目效果明显，全年申报国家和省支持的各类项目105个, 争取中央和省资金7.3亿元，带动总投资52.8亿元。

　　——“双转移”步伐加快。4个省级产业转移工业园实现工业总产值403亿元，增长41%。园区基础设施进一步完善，新投入15.1亿元，新开发土地面积3平方公里，新建标准厂房80万平方米、硬底化道路15公里、供排水管道35.3公里。市政府和佛山禅城（阳东万象）、中山火炬（阳西）产业转移工业园在全省考核中被评为优秀等次。招商引资力度不断加大，在全省率先开展乡贤反哺工程，积极参加各类经贸合作交流会，成功举办第十一届中国（阳江）国际五金刀剪博览会，引进了华润新能源等一批超1000万美元的外资大项目，全市实际利用外资1.53亿美元。农村劳动力转移就业不断加强，组织农村劳动力技能培训3.44万人，新增转移农村劳动力5.57万人，失业人员实现再就业2.96万人，城镇登记失业率2.5%。

　　——城乡建设扎实推进。全面完成了绿道网总体规划编制、海陵岛总体规划修编、城南新区控制性详细规划全覆盖编制，启动了市区长洲岛分区规划调整工作，加快了镇村规划编制进度。城乡交通设施加快建设，阳云高速阳罗段工程和S277线海陵大堤段扩建工程动工，S113线阳春北段改造工程基本完成，阳江港口岸联检综合大楼投入使用，鸳鸯湖环湖路等一批市政项目完工。国家园林城市创建工作正式启动，开展园林绿化“十佳”单位和“百佳”家庭评选活动，建成了市区绿道示范工程和环城河带状公园。建设生态景观林带106公里、碳汇林4.86万亩，累计完成植树造林9.44万亩。“三旧”改造稳妥推进，市区瑞禾路（金源广场）等58个项目顺利实施改造，涉及面积4620亩。节约集约用地水平明显提高，连续13年实现耕地占补平衡。农村生产条件不断改善，超额完成300公里农村公路硬底化建设任务；整治农田5万亩，改善灌溉5.8万亩；争取国家和省补助资金4.5亿元，完成12宗农村饮水安全工程，开展3个农田水利重点县、4个小型农田水利示范镇工程建设，除险加固水库6座，完成海堤加固达标建设25公里，成为全省样板工程。镇村环境整治取得初步成效，建成了镇级垃圾中转站、3325个村庄生活垃圾收集池，奕垌生活垃圾综合处理场成为省首批Ⅰ级生活垃圾无害化填埋场。节能减排和环境污染治理力度进一步加大，漠阳江、马南河综合整治工程取得良好成效；对市第一净水厂等9家国控重点污染源进行实时自动监控；连续8年在全省环保责任考核中获得优良以上等次。

　　——社会事业全面进步。十件民生实事顺利实施。创建教育强市工作扎实开展，完成了中小学校校舍安全工程三年任务，义务教育规范化学校达155所，两阳中学、市一职校分别创建为国家级示范性普通高中和省级示范性中等职业学校，双滘镇、永宁镇成为粤西地区首批省教育强镇。公共文化服务体系逐步完善，每万人拥有公共文化设施面积比上年提升36.2%；广东海上丝绸之路博物馆创建国家二级博物馆和国家4A景区通过省验收。阳江十八子世界音响博物馆入选省十大民间收藏馆。举办了关山月诞辰100周年暨首届关山月文化节、2012南国书香节暨第一届阳江书展、第二届公园艺术节和“阳江好市民”评选等活动。群众体育活动深入开展，每万人拥有社会体育指导员18名，提前实现省下达的群众体育“十二五”规划目标；承办省青少年帆船（帆板）锦标赛，夺团体总分第二名。医疗服务体系建设有效加强，全面完成了基层医疗卫生机构综合改革任务，顺利完成了基本公共卫生服务和重大公共卫生服务项目，新增5个省级临床重点专科。中医强市步伐明显加快，市中医院和阳春市分别被认定为全省中医药强省建设先进单位和全国基层中医药先进单位，新增3个国家农村医疗机构中医特色专科。闸坡镇、合山镇创建为省卫生镇。社会保障机制不断健全，市养老中心顺利推进，城乡低保、农村五保供养和医疗救助水平进一步提高，企业职工和城乡居民养老保险工作扎实开展，覆盖城乡的社会保险体系基本形成。政策性渔业保险工作在全省率先启动。住房保障水平稳步提高，动工兴建了2472套公租房，其中1236套交付使用。稳定物价“三项建设”加快推进，新建成平价商店90家，居民消费价格指数上涨控制在2.6%。农村公益事业“一事一议”奖补工作走在全省前列。扶贫开发“双到”工作成效明显，完成了农村低收入住房困难户4000户泥砖住房改造建设，贫困村集体经济收入年均10.6万元，有劳动能力的贫困户全部实现脱贫。全国人口计生综合改革示范市和婚育新风进万家活动示范市建设稳步推进，在全省率先全面开展免费孕前优生健康检查。妇女儿童十年规划目标基本实现。双拥共建、国防教育、人民防空、民兵预备役工作进一步加强，连续3次获得“全国双拥模范城”称号。市和阳东县残疾人康复中心建设扎实推进。广播电视、新闻出版、民族宗教、外事侨务、供销、打私、气象、地震、科普、档案等工作取得了新成效，老龄、关工委、老促会、红十字、慈善等事业实现了新发展。

　　——社会保持和谐稳定。领导干部定期接访、包案处理和带案下访制度日益健全，市网上信访大厅建成使用，社会矛盾纠纷得到有效化解。平安阳江建设有力推进，社会治安综合治理持续加强，成功打掉了建市以来首例跨国贩毒集团。“三打两建”深入开展，在查办大案要案、斩链挖伞等方面取得突破；启动了社会信用体系和市场监管体系建设试点工作，市场环境明显改善。“安全生产年”活动扎实开展，安全生产形势总体平稳。食品药品专项整治稳步实施，食品药品安全水平不断提高。法律援助体系逐步健全，弱势群体合法权益得到维护。各类防灾减灾应急体系日益完善，预防和处置突发公共事件能力进一步提高。

　　——发展活力持续增强。科学谋划加快发展的重大决策部署，出台了《关于加快海洋经济强市建设的实施意见》等一系列政策措施，有效增强了推动发展的活力。政府职能转变进一步加快，行政审批制度改革持续深化，各部门行政审批以及带有审批性质的管理事项全部纳入清理范围。事业单位分类改革顺利完成，公益服务水平全面提高。城市社区管理体制改革扎实推进，完成了江城区社区公共服务站建设。农村综合改革迈出新步伐，各镇（街道）设立了便民服务中心，完成了农村集体土地确权登记发证工作。投融资体制和地方金融改革稳妥推进，组建了市恒财城市投资控股有限公司，基本完成城信社退市工作，在全省率先建成农业产业链征信系统。社会组织不断发展，新培育社会组织73个。市公共资源交易中心运作逐步完善，实现了市县两级土地、矿业权、工程招标、公共物品采购等集中公开交易。医药卫生体制、居民阶梯电价等其他改革取得新成效。

　　——政府自身建设有效加强。自觉接受市人大及其常委会的工作监督和市政协的民主监督，定期向市人大常委会报告工作，及时向市政协通报情况，广泛听取各民主党派、工商联、无党派人士和工会、共青团、妇联等群众团体的建议意见，办理人大代表议案1件、建议73件，政协提案168件，办复率均为100%。干部培训工作不断加强，各级领导干部引领发展的能力持续提升。效能建设年活动扎实开展，机关作风明显改观。政务公开深入推进，搭建了市网上办事大厅总体框架，实现了与省网上办事大厅双向链接；网络问政工作取得新成效，入选全省网络问政十大经典案例。党风廉政建设责任制全面落实，纠风治乱和案件查处力度加大。审计、监察工作得到加强。“六五”普法深入开展，依法行政工作扎实推进。

　　各位代表，回顾一年来的工作，成绩令人鼓舞。这是市委科学决策、正确领导的结果，是市人大和市政协监督支持、共同努力的结果，是全市人民齐心协力、团结奋斗的结果。在此，我代表市人民政府，向全市人民以及所有关心支持阳江发展的同志们、朋友们，表示衷心的感谢！

　　但我们也清醒地认识到，我市经济社会发展中还存在不少困难和问题。主要是：经济快速发展的基础不牢固，产业规模小，重大项目支撑乏力；资源环境制约日益凸显，自主创新能力薄弱，转变经济发展方式任务繁重；城乡建设基础较差，城镇集聚力和辐射力不强；人民生活水平总体不高，历史遗留问题和新的社会矛盾交织，社会管理难度增大；体制机制障碍需要突破，政府工作人员综合素质有待进一步提高，发展环境仍需改善。对此，我们将采取措施认真加以解决。

　　2013年工作安排

　　2013年是全面贯彻落实十八大精神的开局之年，是实施“十二五”规划承前启后的关键一年，是为全面建成小康社会奠定坚实基础的重要一年。新的一年，我们既要把握机遇，坚定加快发展的信心；更要充分估计形势的复杂性和严峻性，切实增强危机意识和忧患意识，迎难而上，务实工作，努力在幸福追赶的道路上迈出新步伐。

　　今年市政府工作的总体思路是：深入贯彻落实党的十八大、中央经济工作会议、习近平总书记视察广东重要讲话、省委十一届二次全会和市委六届四次全会精神，以科学发展观为指导，紧紧围绕主题主线和 “在加快发展中转变方式、实现幸福追赶”的核心任务，牢牢把握稳中求进的总基调，积极实施“双化”驱动、蓝色崛起、融入珠三角战略，更加注重增强经济综合实力，更加注重推进新型城镇化，更加注重保障和改善民生，更加注重改革创新，努力建设三次产业协调发展的新型工业化城市。

　　全年主要预期目标是：地区生产总值增长13%，规模以上工业总产值增长22%，规模以上工业增加值增长23%，农业总产值增长4.5%，地方公共财政预算收入增长18%，固定资产投资增长18%，社会消费品零售总额增长15%，外贸进出口总额增长8%，实际利用外资增长13%，城镇居民人均可支配收入增长15%，农村居民人均纯收入增长16%，城镇登记失业率控制在 3%以内，居民消费价格涨幅控制在全国平均水平以下，单位生产总值能耗控制在省下达指标范围内。重点抓好以下工作：

　　一、推动经济持续健康发展

　　加快经济发展是当前阳江第一要务。只有推动经济持续健康发展，才能筑牢幸福阳江的物质基础。

　　加大有效投入。今年计划安排重点建设项目60个，总投资1845亿元，年度投资201.3亿元，其中新建项目28个，续建项目26个，投产项目6个。安排预备重点项目43个，总投资965亿元。着力推动低辐射节能玻璃、海螺水泥等一批项目竣工投产，加快阳江核电站、厨邦食品、博德环保陶瓷等一批项目建设步伐，力促阳江抽水蓄能电站、华润水泥二期和阳西白沙湾风电场等一批项目动工，做好中海油天然气发电、大唐华银电厂等一批项目前期工作，科学谋划一批符合国家产业政策和投资导向的项目，努力形成“投产一批、在建一批、开工一批、储备一批”的发展格局。

　　促进社会消费。千方百计增加城乡居民特别是低收入群众收入，促进居民文化、旅游、康体、养老、家政等服务消费。积极引进大型商业连锁企业和专业市场，加快建设华科国际家居生活广场、新都汇时代广场、五金刀剪国际商贸城等城市综合体。加快构建“阳江刀剪”、“阳江海产”、“阳货网城”等电子商务平台。强化监管和服务，坚决打击商业欺诈、制假售假行为，让广大群众放心消费、安全消费。

　　扩大外贸出口。加大外贸出口企业扶持力度，组织企业参加各类经贸活动，扩大主体市场出口规模，开拓俄罗斯、东盟、南美等新兴市场。鼓励自主知识产权和自主品牌产品出口，提高出口产品质量、档次及附加值。推进加工贸易转型升级，大力建设餐厨用品和水产品外贸转型升级示范基地。加快口岸配套设施建设，完善阳江港口岸联检综合大楼管理，进一步优化通关环境。

　　二、加快产业转型升级

　　坚持规划引领，强化产业导向，壮大产业集群，构建现代产业体系，推动三次产业协调发展。

　　加快发展海洋产业。编制海洋经济发展区域布局、产业和项目规划，加强海洋岸线资源管理，统筹开发沿海岸线。推进港口码头泊位等基础设施建设，构筑以阳江港为中心的综合交通运输体系。以高新区港口片区和阳江海洋经济特色产业基地为重要平台，引进一批海洋工程装备制造、船舶制造等临海工业和航运物流等生产性服务业。大力发展现代渔业，扶持发展远洋渔业、深水网箱养殖和健康水产养殖，扎实开展木质渔船改钢质渔船试点。改造升级对岸及溪头渔港，推进南鹏岛整治修复与保护，建设海陵岛国家海洋公园，强化海洋资源有序开发、生态利用和有效保护。

　　突出发展先进制造业。积极发展节能环保、半导体照明、新材料、新能源、生物医药、电子信息、汽车零配件等制造业，大力培育高新技术产业。着力推进年产10万吨纯镍合金产业链精炼加工等项目建设，努力促进一批新兴产业规模化、集群化。

　　提升发展传统产业。以省市共建五金刀剪产业、食品（水产品）加工产业转型升级基地为契机，以原材料、机械装备、工艺技术和工业设计为突破口，强化产学研合作，加快传统产业转型升级步伐。启动改善创新环境五年行动计划，引导企业加强创新载体建设，组建工程技术研究开发中心，提高自主创新能力。深入推进质量强市活动，加强国家刀剪及日用金属工具质量监督检验中心等公共检测服务平台建设。

　　繁荣发展以旅游业为龙头的现代服务业。编制《阳江市滨海旅游发展规划》，明确滨海旅游区域功能。继续完善旅游基础设施，抓好凤凰湖温泉度假区、月亮湾滨海旅游度假区、海陵游艇产业基地等重点旅游项目和一批高星级酒店建设，开展阳春影视旅游文化产业园前期工作。推广网上旅游和旅游电子商务，办好旅游文化节等活动。强化旅游市场监管，提高旅游服务水平。加快发展商贸物流、金融保险、信息科技、商务会展、文化创意等现代服务业，改造提升餐饮、批发零售、社区服务等传统服务业，推动第三产业结构升级。

　　三、提升“双转移”工作水平

　　更加主动承接珠三角的辐射带动，加快与珠三角实现交通、市场、产业和机制对接，提升经济社会发展水平。

　　主动融入珠三角。认真学习珠三角地区先进的发展理念、工作方法和管理经验，不断拓展思路，创新举措，改善环境。积极参与珠三角地区的产业分工，提高关联产业发展水平。着力加强与珠三角地区交通设施的对接，加快推进粤西沿海高速铁路等项目规划建设。认真落实《推动率先基本实现粤港澳服务贸易自由化行动计划》，加强与珠三角内环六市及“湛茂阳”经济圈合作，提高区域合作水平。

　　强力推进园区建设。科学调整和严格执行产业转移园区功能规划，优化园区产业布局。创新园区运作机制，推动产业转移园向经济合作区转变。加大园区融资力度，推进水、电、路、气、排污管网、标准厂房等基础设施以及生活配套设施建设。清理闲置土地，盘活存量土地，用地指标向园区倾斜。完善园区工作考核机制。

　　大力开展招商引资。突出重点，主动联系世界500强和国内大企业大集团，着力引进与核心产业相关联的项目。实施产业链招商、资源招商和以商引商，不断提高招商引资规模和质量，培植规模较大、技术先进、行业领先的产业集群，形成主导产业突出、行业特色明显、产业集聚度高的新型工业基地。

　　着力改善服务环境。建立指导、服务、监管“三位一体”的新型企业投资管理服务体制。坚持领导挂点工业企业制度，推进工业企业集中服务活动常态化。完善中小微企业服务体系，建设专业服务平台，为企业提供法律咨询、政策信息、融资策划等综合服务，营造良好的营商环境。

　　加强劳动力培训转移。加快培训机构基础设施建设，完善镇（街道）人力资源和社会保障服务平台，强化校园与产业园对接，开展订单式、储备式培训，创新培训内容，提高培训质量，让劳务输出由体力型向技能型转变。

　　四、加强农业农村工作

　　围绕“保供增收惠民生、改革创新添活力”目标任务，落实惠农政策，打牢农业农村发展基础，农民收入至少应与城镇居民收入同步增长，并力争超过。

　　大力发展现代农业。提高粮食综合生产能力，促进农业稳定增产。编制现代农业园区发展规划，开展“一镇一园区”创建活动，推动农业生产科技化、规模化和产业化。着力建设南药、水果、花卉、蚕桑等特色优势产品生产基地。推进畜禽标准化规模养殖。发展无公害农产品、绿色食品和有机食品。加快农村土地流转，培育农业龙头企业和农民专业合作社。加强农业实用人才队伍建设。

　　改善农村基础设施。全面铺开海堤加固达标工程建设，推进治洪治涝保安工程、农田水利千宗工程和农村饮水安全工程建设。重点推进漠阳江双捷引水工程拦河闸、阳东县红江拦河闸重建、漠阳江中下游综合治理、四围涝区电排站等工程建设，完善基层水利服务体系。完成34.7万亩高标准基本农田建设任务。抓好农村公益事业“一事一议”财政奖补工作，建设一批农村民心工程，不断完善农村基础设施和公共服务设施。

　　深化农村综合改革。巩固阳春山区县农村综合改革成果，加快全市农村综合改革步伐。以简政放权、机构整合为重点，深化镇级机构改革，完善镇级财政管理体制。完成集体经济年收入20万元以上行政村“政经分离”改革。大力推行村务公开，加强村帐镇管工作，促进村级社会管理民主化、透明化。推进新一轮扶贫开发，实现相对贫困村、贫困户脱贫致富。

　　五、推进新型城镇化

　　深入贯彻落实《广东省促进粤东西北地区地级市城区扩容提质五年行动计划》，把生态文明理念和原则融入城镇化全过程，走集约、智能、绿色、低碳的新型城镇化道路，打造“让本地人自豪，令外地人羡慕”的生态宜居城市。

　　推动市区扩容提质。启动城市总体规划纲要修改。积极拓宽融资渠道，加大城市开发建设力度，加快城南新区等重点区域开发。继续推进市区与阳东县东城、雅韶、红丰镇一体化进程，统筹市区与高新区、海陵岛试验区的空间布局与规划建设，以城市发展引领产业发展，以城市扩容提质辐射、引领区域经济转型升级。深入开展国家园林城市创建活动，加强公园、园林绿化和基础设施建设，全力打通新江东路、东门南路、创业北路、漠江中路、康泰路等市政道路，进一步完善城市功能。抓好“三旧”改造，打造市区松岗山（百利广场）等一批亮点项目。强化城市管理综合执法和环境综合整治，打击各类违法用地、违法建设等行为。加快市区智能交通系统建设，整治违章车辆上路，改善城市交通秩序。将市区周边镇、工业园区纳入公共交通网络，推动城乡客运一体化发展。

　　加强县城和镇村建设。坚持以城带乡，城乡互动，把小城镇建设作为统筹城乡发展、推进新型城镇化的重要平台。努力提升各县城发展水平，改善县城环境，增强吸纳人口和就业能力。加大中心镇培育扶持力度，推动要素资源向中心镇配置、人口向中心镇集聚，使之成为承接城市辐射、带动农村发展的重要平台。完善镇村建设规划，完成462个行政村和399个省级试点村规划编制工作。实施幸福村居五年计划，加快名镇名村示范村和社区基础设施“六个一”工程建设。抓好村庄环境整治，努力实现农村环境“三无五有”目标。

　　建设美丽阳江。科学制订主体功能区规划实施意见。重视生态环境建设，实施最严格水资源管理制度，强化林地、野生动植物、湿地资源和沿海防护林带的保护管理，推进生态景观林带和碳汇林建设，构筑绿色生态屏障，保护阳江的青山绿水、碧海蓝天。大力发展低碳经济，推行清洁生产，努力构建循环经济体系。强化节能减排，加快市区城南、城北和中心镇污水处理厂建设，加强产业转移园污水处理设施的运营管理，抓好马南河综合整治与市区雨污分流管网改造，建设市生活垃圾综合处理环境园、工业固体废物处理中心等环保设施。推进核应急演习、PM2.5监测、环境监测标准化等项目建设。

　　六、致力保障和改善民生

　　统筹社会各方面力量，完善基本公共服务体系，认真办好十件民生实事，努力在学有所教、劳有所得、病有所医、老有所养、住有所居上取得新进展。

　　优先发展教育。扎实推进创建教育强市工作，完成10个教育强镇建设任务，巩固和提高学前教育水平，实现义务教育规范化学校覆盖率85%以上、初中三年保留率96%以上。优化普通高中学校布局，完成两阳中学新校区建设。做强职业技术教育。提升特殊教育办学水平。鼓励和规范民办教育健康发展。加强教师队伍建设。强化学校安全管理，实施新一轮中小学校校舍安全工程，确保新建校舍全部达到抗震设防标准。

　　积极扩大就业。实施更加积极的就业政策，多渠道增加就业岗位。继续实施“南粤高校毕业生就业推进行动”和“三支一扶”等基层服务项目，做好重点群体就业。完善创业相关优惠政策，以创业带动就业。加强劳动保障监察和争议调解仲裁，构建和谐劳动关系。

　　完善社会保障体系。进一步完善统一的城乡居民社会养老、医疗保障体系，实现城乡居民养老保险全覆盖，城乡居民医保参保率巩固在99%以上。落实最低生活保障、教育、医疗、司法等救助制度，实现低保补贴水平自然增长，五保供养标准不低于当地上年度农村人均纯收入的60%。健全住房保障体系，建设公租房1200套，年内竣工500套。落实“米袋子”、“菜篮子”行政首长负责制，推进以平价商店为主体的稳定物价“三项建设”，完善低收入群众临时价格补贴与物价上涨联动机制。

　　加强文化名城建设。办好关山月文化节、南国书香节、第十一届南海（阳江）开渔节等系列活动，丰富城乡群众文体生活。推进“南海Ⅰ号”的整体发掘和保护工作，提升广东海上丝绸之路博物馆的展示水平。做好广东海洋历史博物馆和文化创意（海洋文化）产业园前期工作，打造南粤海洋文化传承基地。大力发展文化产业，塑造漠阳文化品牌。加快市体育中心田径运动场和镇农民体育健身工程建设，完善市、县、镇、村“三馆”（站室）等公共文体设施。

　　大力发展卫生等社会事业。制订中医强市“十二五”规划。加快建设市人民医院新住院大楼，迁建市妇幼保健院，推进基层中医提升工程和医疗卫生机构标准化建设。实施优生惠民工程，完成13个镇级计生服务所达标改造，依法打击“两非”行为，突出治理出生人口性别比偏高问题，稳定低生育水平，促进人口长期均衡发展。加强国防后备力量和人民防空建设，深入开展双拥共建活动。启动市档案方志党史馆建设，编纂出版阳江历史地情文库。做好第三次全国经济普查前期准备工作。实施推动新一轮妇女儿童发展规划。进一步做好广播电视、新闻出版、民族宗教、外事侨务、对台、气象、老龄、残疾人和关工委等工作。

　　创新社会管理。加强平安阳江建设，推进公安社会管理信息化进程，完善社会治安打防控体系，始终保持对违法犯罪活动的严打高压态势。认真做好矛盾纠纷排查化解工作，加大信访积案处理力度，完成各县（市、区）网上信访大厅建设，继续开展领导干部定期接访、带案下访、上门探访和基层巡访活动，进一步畅通信访渠道。扎实开展网络问政工作，提高政府公共服务水平。加强安全生产管理，落实“一岗双责”制度，强化重点行业领域专项整治，推进事故隐患排查治理“一体系三平台”建设，夯实安全生产基础，遏制重特大安全事故发生。强化以食品药品为重点的产品质量安全监管，进一步规范食品药品市场秩序。深入推进“三打两建”工作，抓好重点地区、重点行业“三打”案件的查办，逐步建立完善规范的社会信用体系和市场监管体系。大力培育发展社会组织、中介机构，完善社会管理功能。健全防灾救灾减灾应急体系，提高应对处置突发事件能力和水平。

　　七、加大改革创新力度

　　改革是发展的最大红利。推进重点领域和关键环节的改革，不断突破制约科学发展的体制机制障碍。出台实施行政审批制度改革事项目录，建立健全行政审批监管制度。在海陵岛试验区、高新区和阳江广播电视台、阳江日报社等单位，实施以全员聘用为核心的干部人事制度改革，推进各县（市、区）和镇级行政综合执法改革，进一步完善事业单位分类改革。创新商事登记管理制度改革，放宽市场主体准入限制。加快网上办事大厅建设，7月底前建成省网上办事大厅阳江分厅。深化医药卫生体制改革，有序推进村卫生站实施基本药物制度，以阳东县为试点开展县级公立医院改革。稳妥推进供水等价格改革。

　　八、建设高效廉洁政府

　　进一步加强政府自身建设，增强政府的公信力、执行力和创造力，维护政府良好形象。

　　坚持依法行政。自觉接受人大法律监督和政协民主监督，高度重视接受舆论监督和群众监督。把法治作为理政的基本方式，增强各级领导干部法治观念，严格按照法定权限和程序行使权力、履行职责，依法管理经济社会事务。规范行政处罚自由裁量权，切实做到严格规范、公开文明执法。

　　坚持为民勤政。密切联系群众，做到问政于民、问计于民、问需于民，深入基层、深入一线，了解群众疾苦，倾听群众呼声，尽心尽力为群众排忧解难。强化责任意识，进一步健全政府责任体系，完善行政首长问责制和部门协调配合机制，形成干事创业的良好氛围和工作合力。

　　坚持高效理政。加强公务员队伍能力建设，不断提高工作能力和专业化水平。认真对照政府工作目标，完善精细化、科学化的推进机制，推动各项工作有序有力有效落实。强化督查工作，确保政令畅通。继续开展效能建设年活动，加大治懒治慵治散力度，健全考核激励机制，完善绩效考评制度，提高工作效率。

　　坚持从严治政。严格落实党风廉政建设责任制，推动源头治腐。坚持用制度管权、管事、管人，加强公共财政和经济责任审计监督，强化权力运行监督制约。切实加强作风建设，严格执行中央关于改进工作作风、密切联系群众的八项规定，认真解决群众反映强烈的问题，严肃查处各类违纪违法案件，努力营造风清气正的政务环境。

　　各位代表！阳江发展充满希望，催人奋进。让我们紧密团结在以习近平同志为总书记的党中央周围，高举中国特色社会主义伟大旗帜，在阳江市委的正确领导下，凝聚全市人民的智慧和力量，团结拼搏，开拓进取，为阳江更加幸福美好的明天而努力奋斗！
